# When Diabetes Mimics Scleroderma: Cheiroarthropathy and Neuropathic Digital Erosions in Type 2 Diabetes

**DOI:** 10.7759/cureus.117249

**Published:** 2026-09-30

**Authors:** Joanna Papaioannou, David Wu, Artur Zembowicz, Zachary Schwager

**Affiliations:** 1 Dermatology, Harvard Medical School, Boston, USA; 2 Dermatology, Mass General Brigham, Boston, USA; 3 Dermatopathology, Tufts University School of Medicine, Boston, USA; 4 Dermatology, Lahey Hospital and Medical Center, Burlington, USA

**Keywords:** diabetic cheiroarthropathy, diabetic neuropathy, diabetic stiff hand syndrome, digital erosions, sclerodactyly, scleroderma mimic, systemic sclerosis

## Abstract

Diabetic cheiroarthropathy is an underrecognized complication of diabetes characterized by limited finger joint extension and thickened, waxy skin of the hands. It can clinically mimic several rheumatologic conditions, particularly systemic sclerosis, creating a diagnostic challenge.

We report the case of a 68-year-old man with long-standing type 2 diabetes mellitus who presented with hand stiffness and contractures, scleroderma-like skin thickening, and nonpainful crusted digital erosions, raising concern for systemic sclerosis-associated digital ulceration. He underwent rheumatologic evaluation and received immunosuppressive therapy without improvement in the cutaneous findings, prompting further clinical reassessment. His overall clinical phenotype, including a positive prayer sign, skin thickening confined to the digits, absence of Raynaud phenomenon, normal nailfold capillaries, and negative anti-Scl-70 and anticentromere antibodies, favored diabetic cheiroarthropathy over systemic sclerosis. The digital erosions were considered likely multifactorial, with contributions from unrecognized trauma in the setting of diabetic neuropathy and possible vascular compromise related to peripheral arterial disease and tobacco use.

This case highlights the clinical overlap between diabetic cheiroarthropathy and systemic sclerosis, particularly when digital erosions accompany scleroderma-like hand changes. Recognizing this presentation may help prevent misdiagnosis and unnecessary immunosuppression while directing management toward glycemic control, prevention of repetitive trauma, local wound care, and occupational therapy.

## Introduction

Diabetic cheiroarthropathy, also known as diabetic stiff hand syndrome or limited joint mobility syndrome, often presents with painless finger stiffness, fixed flexion contractures, and thickened, waxy, tight skin of the hands. Patients may also have impaired motor function and decreased grip strength. Characteristic bedside findings include a positive “prayer sign,” in which the patient is unable to fully oppose the palms and fingers, and a positive “tabletop sign,” in which the patient is unable to lay the palms or digits flat on a horizontal surface [1].

One of the principal diagnostic challenges of diabetic cheiroarthropathy is its clinical resemblance to systemic sclerosis, also known as scleroderma. Scleroderma is an autoimmune connective tissue disease characterized by progressive fibrosis of the skin and internal organs, vasculopathy, and immune dysregulation, often accompanied by disease-specific autoantibodies. Common clinical features include sclerodactyly, Raynaud phenomenon, digital ulcers, telangiectasias, and nailfold capillary abnormalities [2,3]. Because diabetic cheiroarthropathy may also present with scleroderma-like skin thickening and limited joint mobility, distinguishing these conditions is important because their management and prognosis differ substantially [3].

Here, we describe a patient with diabetic cheiroarthropathy whose presentation was further complicated by digital erosions, likely related to neuropathic trauma and vascular disease, creating a clinical mimic of systemic sclerosis-associated digital ulceration. This case highlights key clinical and laboratory features that may help differentiate these conditions and avoid unnecessary immunosuppressive therapy.

This case was previously presented at the New England Dermatological Society meeting at Lahey Hospital and Medical Center on April 11, 2026.

## Case presentation

A 68-year-old man was referred to dermatology for a six-month history of skin thickening of the hands and crusted, nonpainful erosions on the right hand.

The patient had initially presented to rheumatology with a several-year history of hand stiffness, swelling, pain, joint contractures, and limited range of motion. Rheumatologic evaluation was negative for rheumatoid factor (RF) and anti-cyclic citrullinated peptide (CCP) antibodies. C-reactive protein (CRP) was elevated at 64.1 mg/L. Hand radiographs demonstrated severe diffuse degenerative changes, particularly involving the first carpometacarpal and interphalangeal joints, with greatest involvement of the fifth proximal interphalangeal joint (Figures 1A, 1B). Subsequent MRI of the left hand demonstrated central erosions of the distal interphalangeal joints and erosive changes at the fifth proximal interphalangeal joint, consistent with erosive hand osteoarthritis (Figure 2A). Additional findings included patchy edema in the bones and mild enhancing synovitis involving the second through fifth metacarpophalangeal joints and the second proximal interphalangeal joint, raising concern for concomitant seronegative inflammatory arthritis (Figure 2B). The patient was started on oral methotrexate at 10 mg/week, which was subsequently increased to 15 mg/week, for suspected rheumatoid arthritis; however, hand pain and swelling continued to progress. A course of prednisone (15 mg/day for three days, followed by 10 mg/day for two days) did not improve his symptoms and was discontinued because of elevated blood glucose levels. After switching to subcutaneous methotrexate at 20 mg/week, he experienced moderate improvement in joint swelling and mild improvement in joint stiffness. However, the skin thickening and erosions persisted, prompting dermatologic evaluation for suspected scleroderma.

**Figure 1:**
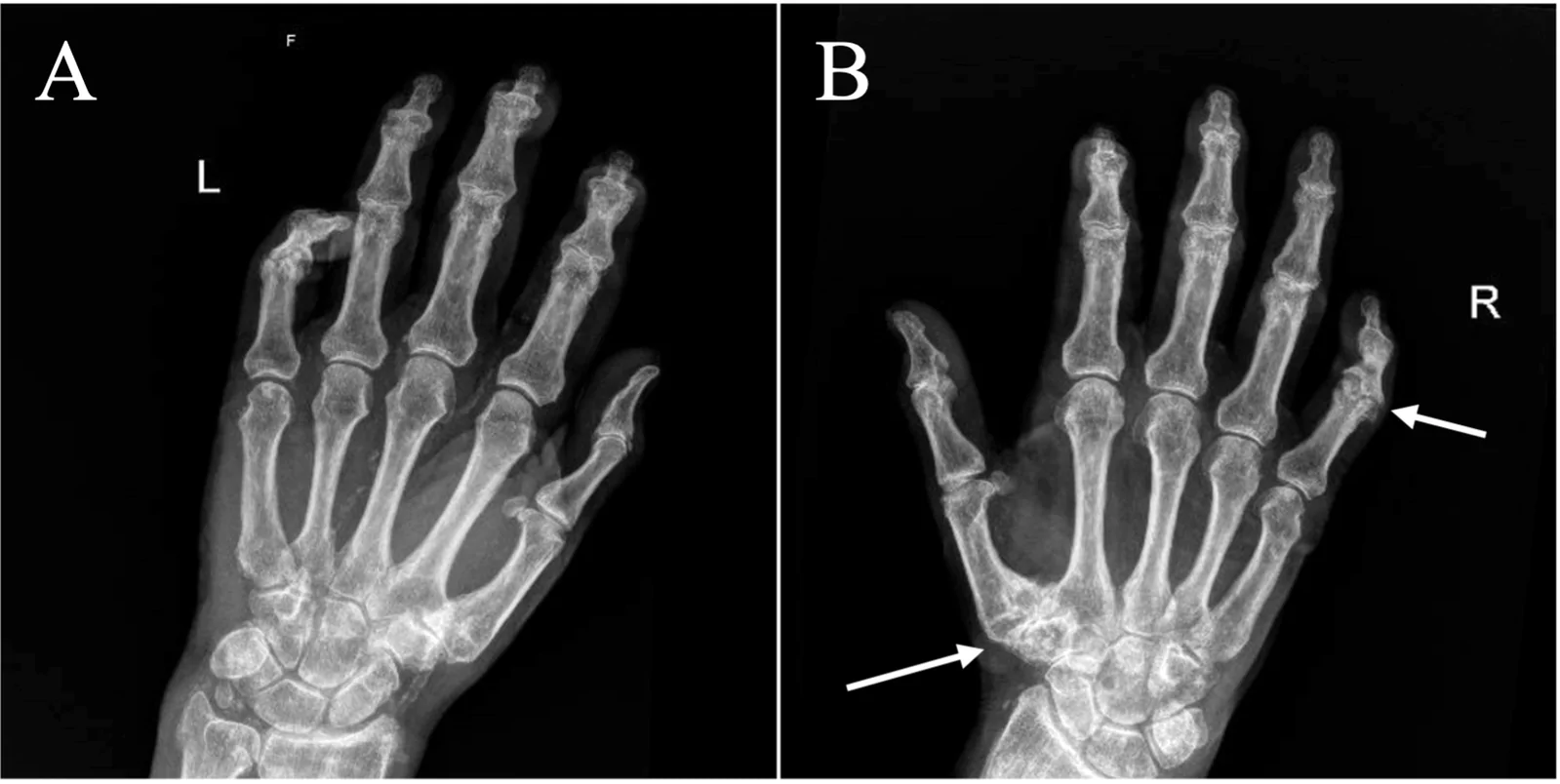
Bilateral hand radiographs demonstrating degenerative changes (A) Left and (B) right hand radiographs demonstrating severe diffuse degenerative changes, with joint space narrowing and articular surface irregularity, most pronounced at the first carpometacarpal and interphalangeal joints. Erosive changes are particularly prominent at the fifth proximal interphalangeal joints bilaterally (arrow in B). Vascular calcifications are present, with no significant soft-tissue swelling. L, left; R, right

**Figure 2:**
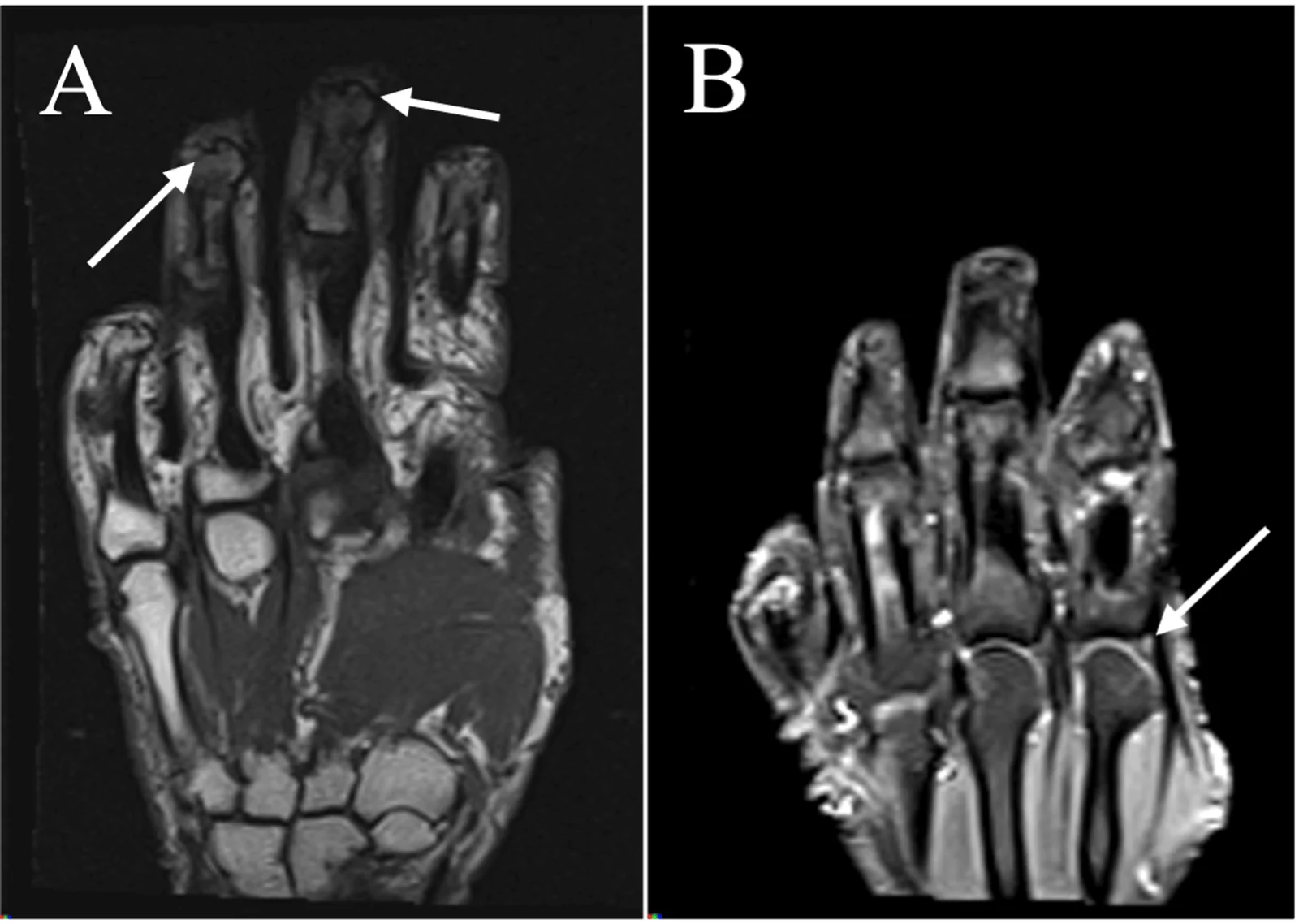
Left hand magnetic resonance imaging demonstrating erosive degenerative changes and mild synovitis (A) Coronal T1-weighted image demonstrating central erosions of the distal interphalangeal joints (arrows). (B) Coronal post-contrast T1-weighted fat-saturated image demonstrating mild enhancing synovitis involving the second through fifth metacarpophalangeal joints and the second proximal interphalangeal joint, with an arrow highlighting synovial enhancement at the second metacarpophalangeal joint.

His medical history was notable for type 2 diabetes mellitus with a hemoglobin A1c of 8.7% (reference range, 4.6%-5.6%), peripheral artery disease, peripheral neuropathy involving the extremities, foot and sacral ulcerations, atrial fibrillation on anticoagulation, and ongoing tobacco use. Upper-extremity vascular perfusion had previously been assessed using pulse volume recordings and segmental limb pressures, which demonstrated digital-brachial indices of 0.72 on the right and 0.68 on the left, consistent with mildly reduced digital perfusion. There was no family history of autoimmune disease. The patient reported frequently injuring his hands and feet without noticing. He denied Raynaud phenomenon.

On physical examination, both hands demonstrated moderate diffuse nonpitting edema, skin tightening, and areas of pallor over the digits. Skin thickening of the dorsal fingers extended proximally beyond the proximal interphalangeal joints to the level of, but not proximal to, the metacarpophalangeal joints. Several dry, superficial, crusted erosions were present on the second and third digits of the dorsal right hand (Figures 3A, 3B), with a few additional erosions on the right forearm. Heberden and Bouchard nodes, stiffness, and limited range of motion were noted. He was able to make a fist but was unable to fully oppose his palms and fingers, indicating a positive prayer sign. Nailfold capillaries were examined by dermoscopy and showed no dilation, dropout, or other abnormalities. There were no facial telangiectasias or other skin abnormalities elsewhere on the body.

**Figure 3:**
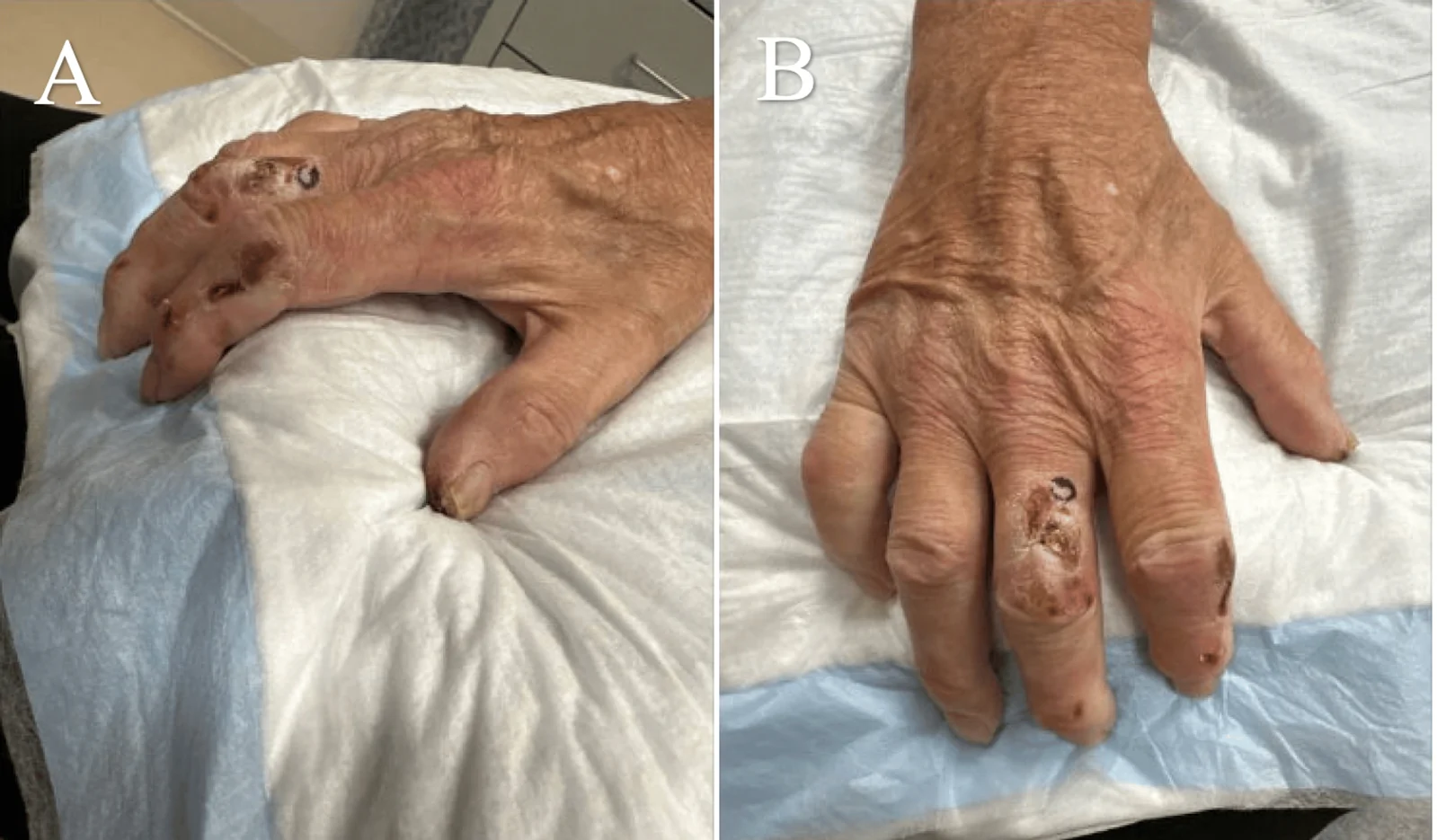
Clinical appearance of the right hand at the initial dermatology presentation (A, B) Dorsal views of the right hand after eight weeks of subcutaneous methotrexate, demonstrating limited finger extension and crusted erosions over the second and third digits.

Laboratory evaluation showed a low-titer positive antinuclear antibody (ANA) at 1:40 (reference range, negative at <1:40). ANA testing by indirect immunofluorescence demonstrated a mitotic intercellular bridge pattern and a nuclear speckled pattern. Anti-Scl-70, anticentromere, and anti-RNA polymerase III antibodies were negative. Antineutrophil cytoplasmic antibodies (ANCA) and cryoglobulins were also negative. A 24-hour urine collection for porphyrins was negative. A recent CT scan of the abdomen and pelvis, which included views of the lung bases, showed no evidence of interstitial lung disease.

A punch biopsy from the third digit of the right hand demonstrated hyperkeratosis and acanthosis with a superficial dermal acute and chronic inflammatory infiltrate and telangiectasias (Figure 4). The findings were described as subtle and nonspecific and were considered most consistent with inflammatory changes adjacent to an ulceration. No fungi were identified on periodic acid-Schiff with diastase (PAS-D) staining, and there was no evidence of increased dermal mucin or excess collagen deposition.

**Figure 4:**
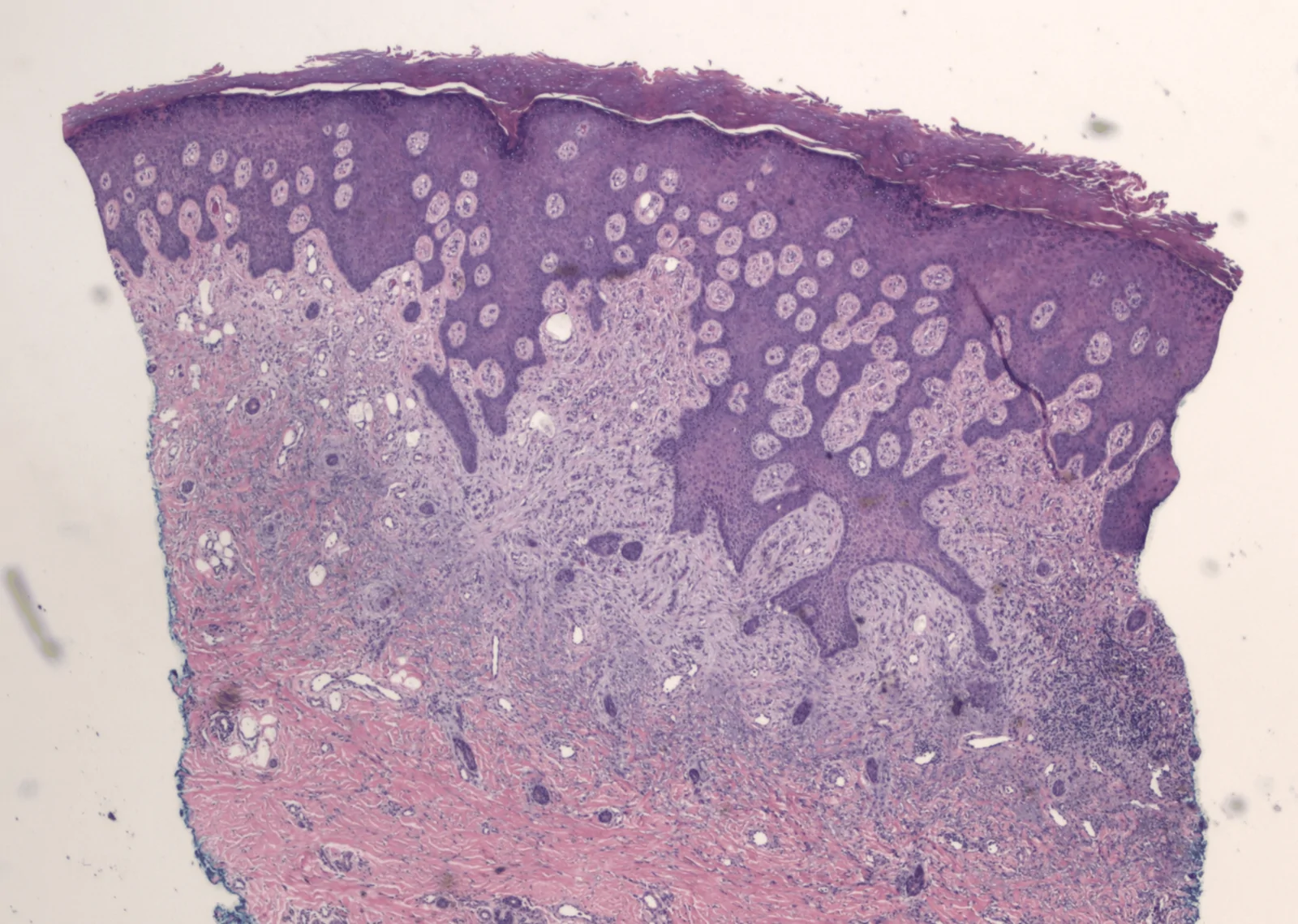
Histopathologic findings from the third digit of the right hand Punch biopsy specimen from the third digit of the right hand demonstrating hyperkeratosis and acanthosis with a superficial dermal inflammatory infiltrate (H&E, ×40). The findings were nonspecific and most consistent with inflammatory changes adjacent to an ulceration.

The overall clinicopathologic context supported a diagnosis of diabetic cheiroarthropathy, complicated by digital erosions likely caused by neuropathic trauma or impaired digital perfusion. Topical mupirocin 2% ointment was prescribed for application three times daily for seven days. At the six-week dermatology follow-up, the digital erosions had improved, although skin thickening and restricted hand mobility persisted (Figures 5A, 5B). Improved glycemic control and occupational therapy were recommended as part of ongoing management. 

**Figure 5:**
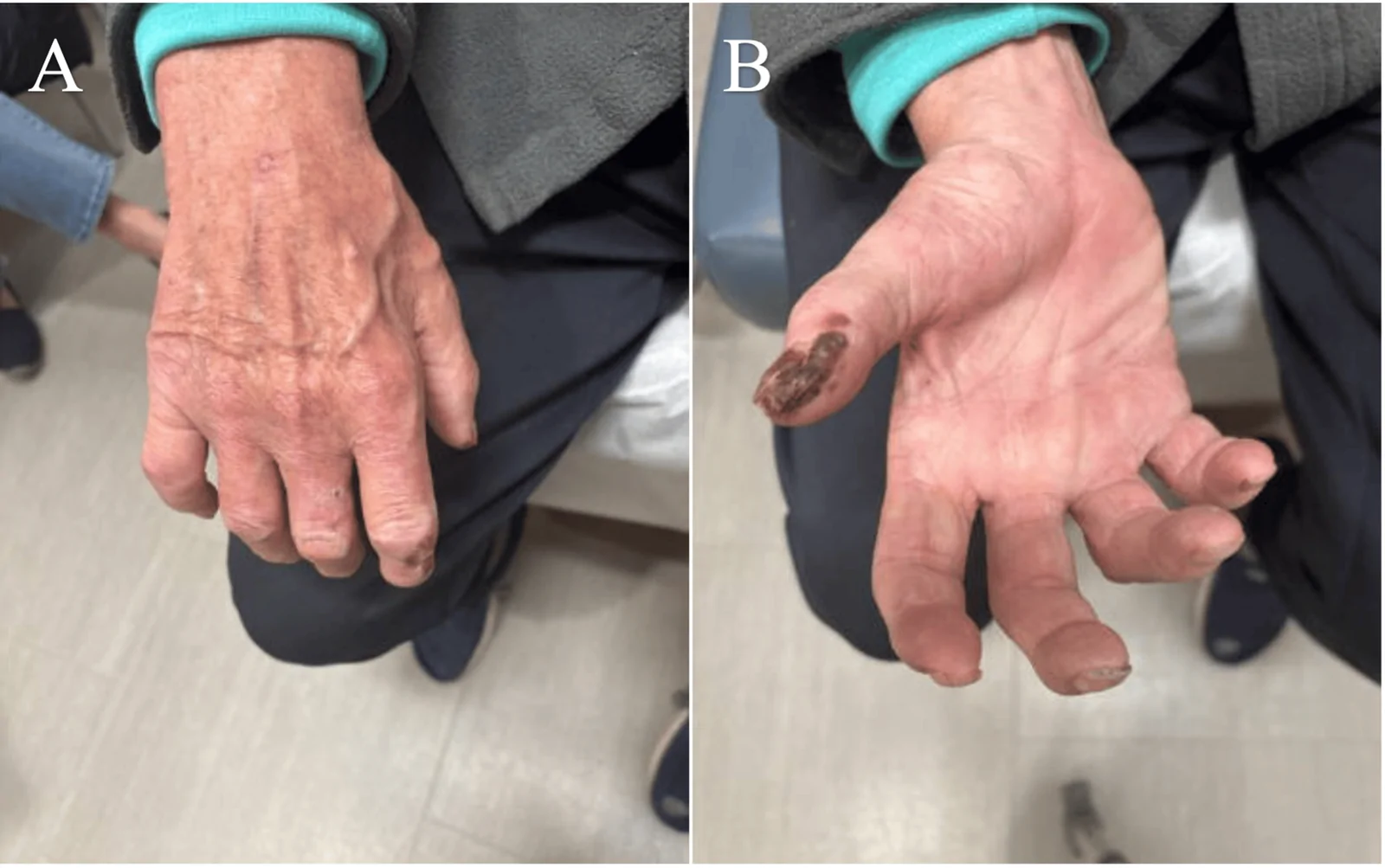
Clinical appearance of the right hand at dermatology follow-up (A) Dorsal view of the right hand at six-week follow-up after mupirocin treatment, showing improved digital erosions. (B) Palmar view of the right hand at six-week follow-up, demonstrating persistent limitation of finger joint extension.

## Discussion

The clinical presentation of diabetic cheiroarthropathy can closely resemble systemic sclerosis, as both conditions may present with sclerodactyly-like skin thickening and reduced hand mobility [3]. However, systemic sclerosis is often accompanied by Raynaud phenomenon, nailfold capillary abnormalities, disease-specific autoantibodies, and, in many patients, extracutaneous internal organ involvement. In our patient, the absence of Raynaud phenomenon, normal nailfold capillaries, negative anti-Scl-70 and anticentromere antibodies, and skin thickening that did not extend proximal to the metacarpophalangeal joints did not definitively exclude systemic sclerosis; the overall clinical phenotype favored diabetic cheiroarthropathy over systemic sclerosis. Under the 2013 American College of Rheumatology/European League Against Rheumatism (ACR/EULAR) systemic sclerosis classification criteria, the patient scored four points for sclerodactyly, below the nine-point threshold for classification [4]. The nonspecific findings on skin biopsy, along with the absence of increased dermal mucin or collagen deposition, neither excluded systemic sclerosis nor provided definitive histopathologic support for diabetic cheiroarthropathy.

The patient's digital erosions further complicated the diagnostic evaluation because ischemic digital ulceration is a well-recognized manifestation of systemic sclerosis resulting from severe vasculopathy [5]. In this patient, however, severe peripheral diabetic neuropathy with frequent unrecognized trauma, together with vascular contributions from peripheral artery disease and ongoing tobacco use, provided plausible alternative explanations for the erosions [6,7]. Decreased digital perfusion associated with peripheral artery disease and tobacco use may also have impaired wound healing [7,8]. Given the overall clinical presentation, the digital erosions were therefore considered likely to reflect a combination of neuropathic trauma and vascular disease rather than systemic sclerosis-associated ischemia.

Erosive hand osteoarthritis and seronegative inflammatory arthritis remained relevant potential concomitant conditions in this patient. Improvement in joint swelling following methotrexate suggested an inflammatory component, potentially related to seronegative inflammatory arthritis [9]. The limited improvement in joint stiffness suggested a dual contribution from both inflammatory arthritis and diabetic cheiroarthropathy, the latter of which would not be expected to improve with immunosuppressive therapy [1]. The patient’s hand pain was more consistent with osteoarthritis and inflammatory arthritis, whereas the positive prayer sign and persistent restriction in joint mobility were characteristic of diabetic cheiroarthropathy [1,9,10]. The persistence of scleroderma-like skin thickening despite treatment with methotrexate and prednisone, together with the absence of characteristic systemic sclerosis features, further supported diabetic cheiroarthropathy as the primary explanation for the patient’s cutaneous findings [3].

The pathogenesis of diabetic cheiroarthropathy is thought to involve the nonenzymatic glycation of long-lived proteins such as collagen, resulting in the formation of advanced glycation end products. These products promote abnormal cross-linking between adjacent collagen molecules, reducing their susceptibility to proteolytic degradation and contributing to the progressive accumulation of extracellular matrix in periarticular tissues [11-13]. These changes may reduce tissue elasticity and contribute to limited joint mobility [11]. Additionally, diabetic microangiopathy affecting the skin and small blood vessels may contribute to chronic low-grade tissue ischemia and fibrosis, resulting in thickened, tight, and less pliable skin of the hands [1].

The reported prevalence of diabetic cheiroarthropathy varies widely, with estimates ranging from 16% to 54% among patients with type 2 diabetes mellitus [14,15]. In patients with type 1 diabetes mellitus, one study reported a prevalence of 66% [16]. Diabetic cheiroarthropathy is associated with longer diabetes duration, poor glycemic control, and other microvascular complications, including diabetic retinopathy and neuropathy [1,3,11,15,17]. Despite its prevalence, diabetic cheiroarthropathy remains underrecognized in routine clinical practice [10].

Although there is no disease-specific therapy for diabetic cheiroarthropathy, management focuses on optimizing glycemic control and preserving hand function through physical or occupational therapy [1].

## Conclusions

Diabetic cheiroarthropathy should be considered in patients with long-standing diabetes who present with scleroderma-like hand thickening and limited joint mobility, even when atypical features such as digital erosions are present. In patients with diabetic neuropathy, recurrent unrecognized trauma, potentially compounded by vascular disease, may produce erosions that closely resemble systemic sclerosis-associated digital ulceration, further complicating the distinction between these conditions. Assessment of the overall clinical phenotype, including the proximal extent of skin thickening, presence of Raynaud phenomenon, nailfold capillary abnormalities, systemic sclerosis-specific autoantibodies, and extracutaneous manifestations, can help clarify the diagnosis. Early recognition of diabetic cheiroarthropathy may help prevent unnecessary immunosuppressive therapy while directing management toward glycemic optimization, wound prevention and care, and rehabilitation to preserve hand function.

## References

[1] Hill NE, Roscoe D, Stacey MJ, Chew S (2019). Cheiroarthropathy and tendinopathy in diabetes. Diabet Med.

[2] Jerjen R, Nikpour M, Krieg T, Denton CP, Saracino AM (2022). Systemic sclerosis in adults. Part I: clinical features and pathogenesis. J Am Acad Dermatol.

[3] Ferreli C, Gasparini G, Parodi A, Cozzani E, Rongioletti F, Atzori L (2017). Cutaneous manifestations of scleroderma and scleroderma-like disorders: a comprehensive review. Clin Rev Allergy Immunol.

[4] van den Hoogen F, Khanna D, Fransen J (2013). 2013 classification criteria for systemic sclerosis: an American College of rheumatology/European league against rheumatism collaborative initiative. Ann Rheum Dis.

[5] Hughes M, Allanore Y, El Aoufy K, Denton CP, Khanna D, Krieg T, Matucci-Cerinic M (2021). A practical approach to the management of digital ulcers in patients with systemic sclerosis: a narrative review. JAMA Dermatol.

[6] Wang C, Lv L, Wen X (2010). A clinical analysis of diabetic patients with hand ulcer in a diabetic foot centre. Diabet Med.

[7] Jeffcoate W, Boyko EJ, Game F, Cowled P, Senneville E, Fitridge R (2024). Causes, prevention, and management of diabetes-related foot ulcers. Lancet Diabetes Endocrinol.

[8] Silverstein P (1992). Smoking and wound healing. Am J Med.

[9] Di Matteo A, Bathon JM, Emery P (2023). Rheumatoid arthritis. Lancet.

[10] Persad-Paisley EM, Lee C, Bhatt RA (2024). Understanding diabetic cheiroarthropathy: a focus on clinical presentation. J Surg Case Rep.

[11] Buckingham BA, Uitto J, Sandborg C (1984). Scleroderma-like changes in insulin-dependent diabetes mellitus: clinical and biochemical studies. Diabetes Care.

[12] Brownlee M (1992). Glycation products and the pathogenesis of diabetic complications. Diabetes Care.

[13] Goh SY, Cooper ME (2008). The role of advanced glycation end products in progression and complications of diabetes. J Clin Endocrinol Metab.

[14] Akanji AO, Bella AF, Osotimehin BO (1990). Cheiroarthropathy and long term diabetic complications in Nigerians. Ann Rheum Dis.

[15] Lu YC, Wang PW, Liu RT, Tung SC, Chien WY, Hung SL, Chen M (1993). Limited joint mobility of the hand: prevalence and relation to chronic complications in non-insulin-dependent diabetes mellitus patients. J Formos Med Assoc.

[16] Larkin ME, Barnie A, Braffett BH (2014). Musculoskeletal complications in type 1 diabetes. Diabetes Care.

[17] Paul A, Gnanamoorthy K (2023). The association of diabetic cheiroarthropathy with microvascular complications of type 2 diabetes mellitus: a cross-sectional study. Cureus.

